# Development of a sheep model of knee osteoarthritis and preliminary magnetic resonance–guided focused ultrasound evaluation

**DOI:** 10.1002/ame2.70266

**Published:** 2026-07-06

**Authors:** Xuejun Du, Andrew Spiteri, Arik Hananel, Abhijit Dighe, Richard J. Price, Xinlin Yang, Quanjun Cui

**Affiliations:** ^1^ Department of Orthopaedic Surgery University of Virginia Charlottesville Virginia USA; ^2^ UVA Focused Ultrasound Center University of Virginia Charlottesville Virginia USA; ^3^ Department of Biomedical Engineering University of Virginia Charlottesville Virginia USA

**Keywords:** magnetic resonance imaging, magnetic Resonance‐Guided Focused Ultrasound, osteoarthritis, sheep model

## Abstract

Knee osteoarthritis (KOA) is a prevalent and disabling disease with limited nonsurgical options for pain management. Magnetic resonance–guided focused ultrasound (MRgFUS) is a noninvasive technique for targeted thermal ablation and has emerged as a potential therapy for pain control. Large‐animal models that better replicate human joint anatomy are needed for translational evaluation. Although monosodium iodoacetate (MIA) is widely used to induce KOA in small animals, its application in sheep remains limited. KOA was induced in the right knees of nine sheep using intra‐articular MIA injections on days 8 and 29, with contralateral knees serving as controls. Animals were monitored for 12 weeks with serial behavioral and radiographic assessments. MRgFUS treatment was performed between weeks 6 and 8 after the first MIA injection. At the study endpoint, knee joints were obtained for macroscopic and histological evaluation. MIA‐treated knees demonstrated radiographic features of KOA, including joint space narrowing and osteophyte formation. Gross and histological analyses confirmed cartilage degeneration, with surface erosion, reduced proteoglycan staining, and disrupted cartilage architecture. Despite consistent structural changes, only two animals developed persistent pain‐related behaviors. In these animals, MRgFUS treatment was associated with improved pain scores and activity levels. No treatment‐related increases in pain, functional impairment, or tissue damage were observed. Intra‐articular MIA injection produced a reproducible structural model of KOA in sheep. MRgFUS was well tolerated and exhibited preliminary potential for pain relief, supporting further evaluation in larger controlled studies.

## Graphical abstract

This study was the first to induce knee osteoarthritis (KOA) by administering monoiodoacetate to sheep to develop a novel animal model of KOA. Radiographic, gross, and histological changes consistent with KOA developed in all sheep; however, only two sheep developed behavioral signs of KOA pain. Magnetic resonance–guided focused ultrasound (MRgFUS) was used to ablate the innervation of the knee joint, reducing behavioral signs of pain in the sheep demonstrating pain without damaging off‐target tissues.

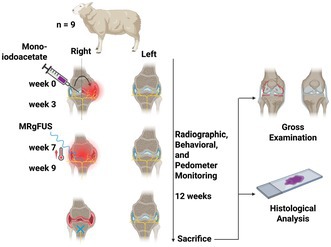

## 1 INTRODUCTION

Osteoarthritis (OA) is a degenerative disease characterized by progressive cartilage degradation, subchondral bone remodeling, and whole‐joint pathology.^1^ OA is a significant and growing global health burden. Globally, the prevalence of OA is 7.6%, a figure that has more than doubled since 1990.^2^ In the United States, one in five adults report OA symptoms, with one in three people who are obese developing symptoms during their lifetime.^3^ Knee osteoarthritis (KOA) is the most common form of the disease and a leading cause of disability.^4^ KOA progresses insidiously; as the disease progresses, pain with activity worsens and joint mobility becomes increasingly limited.^5^

KOA can be managed nonpharmacologically through physical therapy, occupational therapy, and weight management, as well as pharmacologically through topical nonsteroidal anti‐inflammatory drugs (NSAID) and intra‐articular glucocorticoid injections.^6^ Treatment is aimed at decreasing pain and improving joint function. Whereas NSAIDs can provide relief from disease‐related pain, oral NSAIDs may be contraindicated in patients with comorbidities due to their numerous toxicities (gastrointestinal, renal, central nervous system, etc.).^7^ Intra‐articular glucocorticoid injections have been shown to provide pain relief, particularly in patients with more severe symptoms; however, current recommendations limit their frequency due to concerns regarding accelerated cartilage loss.^8^ Delaying or preventing the need for knee replacement surgery through these nonsurgical treatments is important due to the limited lifespan of prosthetic joints and the risks inherent to surgery.^5^, ^9^ Therefore, there is a need to develop new noninvasive interventions to better manage KOA pain.

Animal models provide clinically relevant ways to study the efficacy of novel and established treatments for OA. Numerous methods exist for inducing experimental disease in animals, including spontaneous models, mechanical insult or surgically induced joint instability, and extracellular matrix damage or disruption of chondrocyte metabolism through agents such as monosodium iodoacetate (MIA), collagenase, and steroids.^10^, ^11^, ^12^ MIA‐induced OA is achieved through intra‐articular injection of MIA, a glycolysis inhibitor that disrupts chondrocyte metabolism. The injected MIA disrupts chondrocyte metabolism causing cell death and cartilage degradation.^13^ This approach induces rapid and reproducible structural changes, making it a useful model for evaluating treatment efficacy and pain‐related outcomes.^14^

Both small‐animal models (e.g., mice, rats, and guinea pigs) and large‐animal models (e.g., dogs, sheep, and horses) are used in the study of OA.^10^, ^11^ Whereas small‐animal models have the advantages of easier housing and lower costs, large‐animal models better replicate the joint anatomy of humans and allow more tissue to be recovered for analysis.^10^, ^11^, ^15^ Among these, sheep provide a particularly relevant model because the ovine stifle joint closely resembles the human knee.^16^, ^17^ Most established ovine models are surgically induced, typically involving partial or total meniscectomy. Anterior (or cranial) cruciate ligament transection alone is reported to induce limited cartilage damage in sheep.^16^ Reports of chemically induced diseases using MIA in sheep remain limited. Therefore, we sought to develop a novel MIA‐induced model in sheep and evaluate the associated morphological and histological changes after intra‐articular injection.

Magnetic resonance–guided focused ultrasound (MRgFUS) uses magnetic resonance imaging (MRI) and thermal monitoring to precisely target and thermally ablate tissue using high‐intensity focused ultrasound.^18^ Numerous features make MRgFUS clinically appealing, including its precision and its safety as a noninvasive procedure that does not utilize ionizing radiation.^19^ For these reasons, MRgFUS is currently being investigated for clinical applications in numerous fields, including functional neurosurgery,^20^ oncology,^21^ neuropathic pain,^22^ psychiatry,^23^ and OA.^24^, ^25^, ^26^, ^27^, ^28^ For management of arthritis pain, MRgFUS has been studied in patients with lumbar facet joint disease^24^, ^28^ and knee involvement.^25^, ^26^, ^27^ However, preclinical validation in large‐animal models that closely mimic human joint anatomy remains limited.

In this study, we investigated the use of MRgFUS to thermally ablate periarticular sensory nerve fibers for pain modulation in a sheep model of MIA‐induced OA. Treatment was directed to the periarticular region at the interface between the distal femur and the articular cartilage to target periarticular nerve structures supplying the knee joint. We evaluated the structural effects of MIA‐induced KOA and explored the feasibility and safety of MRgFUS treatment in this model. We hypothesized that intra‐articular MIA injection would induce characteristic structural changes and that MRgFUS could be safely applied to modulate associated pain.

## 2 MATERIALS AND METHODS

### 2.1 Animals

Dorset–Merino white sheep were provided by the Biological Resources Center at the University of Virginia. The animals were housed at a temperature of 21–23°C and a relative humidity of 40%–60%. The sheep were allowed free access to water, and they were fed a commercial sheep diet.

### 2.2 Preparation of MIA solution

MIA (0.3 g, Merck, Nogent‐sur‐Marne, France) was dissolved in 50 mL of sterile phosphate‐buffered saline (pH 7.4, Sigma‐Aldrich, Germany) to yield a stock solution. Each intra‐articular injection consisted of 4 mL of this solution, corresponding to 24 mg of MIA per injection.

### 2.3 Animal model of KOA

This study was approved by the Institutional Animal Care and Use Committee at the University of Virginia (protocol no. 3933). Nine adult Dorset–Merino sheep (average weight: 70 kg, ~2 years old) were used. All sheep were housed for 1 week prior to procedures to establish baseline activity and intake. Prior to anesthesia, food was withheld for 24 h and water for 8–12 h.

On day 8, animals were anesthetized using intravenous ketamine (15 mg/kg, Ceva Tiergesundheit) and medetomidine (0.01 mg/kg, Domitor, Pfizer). The right knee joint was shaved, sterilized, and injected intra‐articularly using a landmark‐based approach targeting the joint space. Each animal was administered 4 mL of MIA solution (24 mg) on day 8 (first of week 2) and a second identical injection on day 29 (first of week 5). The dose was selected based on prior MIA dose–response studies and adapted for large‐animal use based on scaling considerations.^29^ The contralateral knee served as an internal control, with the limitation of potential compensatory loading effects considered in interpretation.

Animals were monitored for 12 weeks with serial behavioral, clinical, and radiographic assessments. Behavioral assessments were performed weekly.

### 2.4 Behavioral and functional assessment

A semiquantitative scoring system was used to assess OA‐associated pain and functional impairment. The scoring system included (1) standing weight bearing (0: continuous weight bearing, 1: intermittent weight bearing, 2: non‐weight bearing); (2) walking/gait (0: normal, 1: intermittent weight bearing, 2: toe‐touching/partial weight bearing, 3: non‐weight bearing); (3) physical examination (limb posture, joint swelling, resistance to manipulation); and (4) activity level: assessed qualitatively and supplemented by pedometer measurements in a subset of animals (*n* = 3). Pedometers were used to quantify daily step counts as an objective measure of activity.

Scores were analyzed by domain rather than as a composite score. This scoring system was adapted from commonly used large‐animal lameness assessments but has not been formally validated, which is acknowledged as a limitation.

### 2.5 MRgFUS procedure

MRgFUS treatments were performed between weeks 6 and 8 after the first MIA induction using a clinical MRgFUS system integrated with a clinical MRI scanner. Real‐time MRI guidance and proton resonance frequency shift–based MR thermometry were used for treatment planning, targeting, and thermal monitoring.

Animals were positioned supine with the treated knee centered over the ultrasound transducer and acoustically coupled using a degassed water bath. High‐resolution anatomical MR images (e.g., T1‐ and T2‐weighted sequences) were obtained for treatment planning and identification of target structures.

The region of treatment (ROT) was defined as the periarticular interface between the distal femur and the articular cartilage, corresponding to the anatomical distribution of genicular nerve branches supplying the knee joint. Targeting was performed using multiplanar MRI guidance based on established anatomical landmarks for periarticular innervation.

Each treatment consisted of a series of discrete sonications. The planned sonication parameters were as follows: frequency: 1 MHz, acoustic power: 85 W, duration: 20 s per sonication, and energy delivery: ~1700 J per sonication. For each ROT, 11 sonications were planned to ensure adequate spatial coverage of the target region. In representative treatments, delivered energy closely matched planned parameters (e.g., 1698 J delivered vs. 1700 J planned for an individual sonication).

Real‐time MR thermometry was performed during each sonication to monitor temperature elevation within the target region. The maximum recorded temperature reached ~64°C, consistent with thresholds for thermal ablation of periarticular sensory nerve fibers. Temperature maps were reviewed continuously to confirm accurate energy deposition and to minimize off‐target heating.

The procedure was designed to achieve focal thermal ablation of periarticular sensory nerve fibers (including branches of the genicular nerves) while preserving the adjacent bone, cartilage, and surrounding soft tissues.

### 2.6 Macroscopic and histological evaluation

At 11 weeks after the initial MIA injection, animals were euthanized by lethal injection of phenobarbitone, and knee joints were harvested for analysis. All evaluations were performed in a blinded manner by at least two independent observers. For macroscopic (gross) evaluation, the articular surfaces of the femoral condyles and tibial plateau were exposed and visually inspected for cartilage degeneration, including surface fibrillation, erosion, ulceration, and osteophyte formation. Representative images were recorded.

For histological analysis, joint tissues were collected from weight‐bearing regions of the medial femoral condyle and tibial plateau, fixed in 10% neutral‐buffered formalin, and decalcified in ethylenediaminetetraacetic acid (EDTA) for 4 weeks. Samples were subsequently embedded in paraffin, and 6‐μm thick sections were prepared. Sections were stained with hematoxylin and eosin (H&E) to assess general morphology and with Safranin O/Fast Green to evaluate cartilage proteoglycan content.

Histological assessment was performed descriptively, focusing on the characteristic features of cartilage degeneration, including surface irregularity, matrix loss, reduced proteoglycan staining, and changes in chondrocyte morphology. A formal semiquantitative scoring system was not applied due to the exploratory nature of this large‐animal study.

### 2.7 Statistical analysis

Due to the exploratory and pilot nature of this study and the small sample size, formal statistical hypothesis testing was not performed. Data are presented descriptively. No sample size calculation was conducted a priori, and the study was designed to assess feasibility and safety.

## 3 RESULTS

### 3.1 Intra‐articular injection of MIA resulted in radiographic and gross pathological features consistent with KOA

**FIGURE 1:**
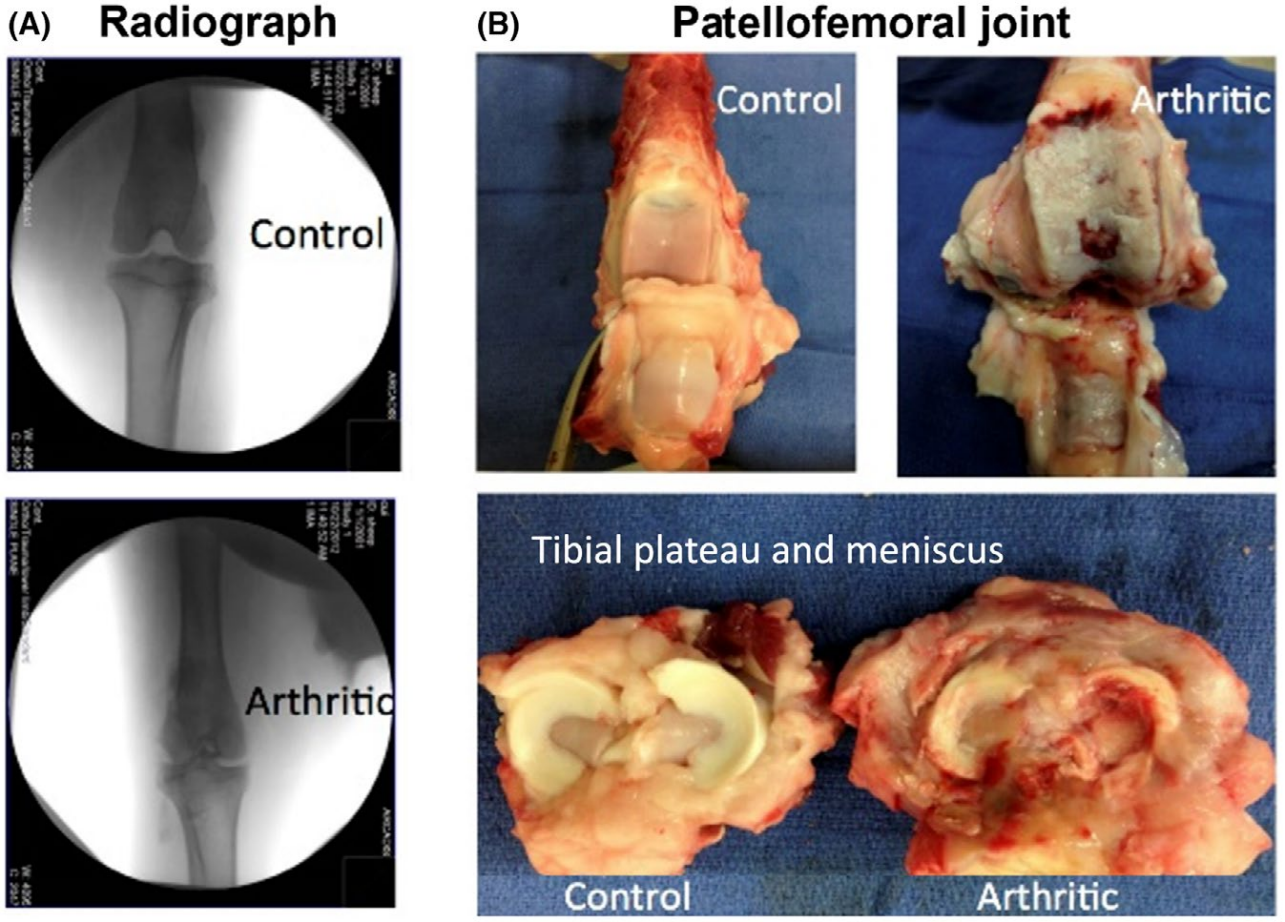
Knee osteoarthritis (KOA) induced by monosodium iodoacetate (MIA) in a sheep model. (A) Representative radiographs of control (left) and MIA‐treated (right) knee joints. (B) Representative gross pathological specimens of the patellofemoral joint and tibial plateau with meniscus of control and MIA‐treated knee joints.

Radiographic and gross pathological changes consistent with osteoarthritic degeneration were observed in MIA‐treated joints. Radiographic analysis demonstrated joint space narrowing and osteophyte formation in the treated knees, whereas control joints showed no radiographic evidence of KOA^30^ (Figure 1A). Gross examination of dissected knee joints revealed pronounced cartilage degeneration in MIA‐treated joints. The articular cartilage surface appeared irregular, with areas of fibrillation and erosion. In contrast, control joints exhibited a smooth, polished, and intact cartilage surface. Examination of the tibial plateau and meniscus further demonstrated extensive chondral erosion in MIA‐treated joints, with exposure of the subchondral bone, whereas no such changes were observed in control joints (Figure 1B).

### 3.2 Intra‐articular injection of MIA induced histological changes consistent with KOA

**FIGURE 2:**
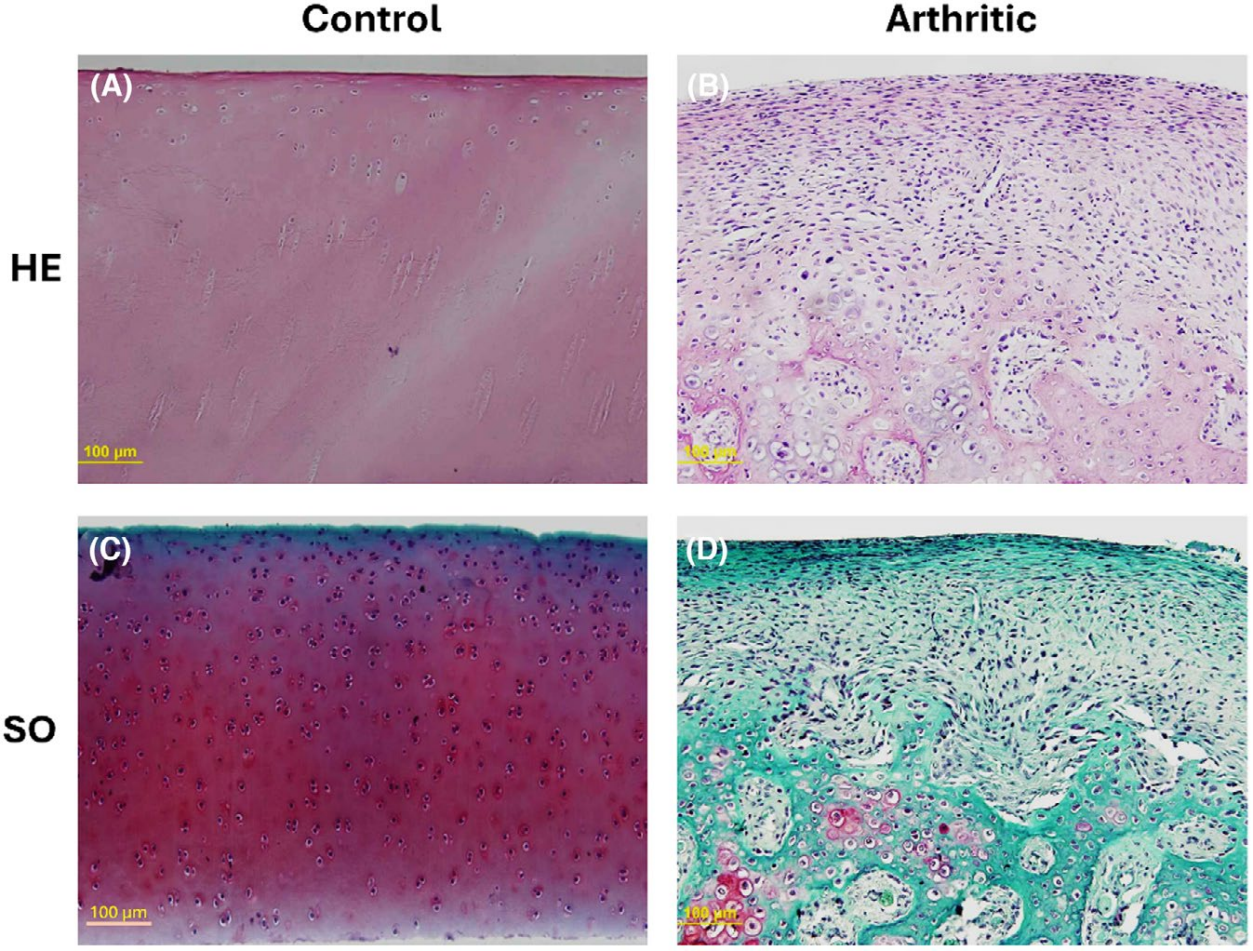
Representative (A, B) H&E (hematoxylin and eosin)–stained and (C, D) Safranin O–stained specimens of cartilage and subchondral bone of control and monosodium iodoacetate (MIA)–treated knees in sheep.

H&E and Safranin O/Fast Green staining of cartilage and subchondral bone sections demonstrated pronounced cartilage degeneration in MIA‐treated joints. H&E staining revealed the disruption of normal cartilage architecture, including disorganization of chondrocytes, loss of tidemark integrity, and thinning of the cartilage layer. In contrast, control joints exhibited well‐organized cartilage structure, with a columnar arrangement of chondrocytes, a clearly defined tidemark, and a thick cartilage layer overlying the subchondral bone surface. These features were significantly altered in MIA‐treated joints, consistent with advanced osteoarthritic changes^31^ (Figure 2A,B). Safranin O/Fast Green staining demonstrated strong proteoglycan staining in control cartilage, whereas MIA‐treated joints exhibited significantly reduced staining intensity, indicating loss of proteoglycan content and cartilage degradation (Figure 2C,D).

### 3.3 KOA‐associated pain was observed in a subset of animals and improved after MRgFUS treatment

**FIGURE 3:**
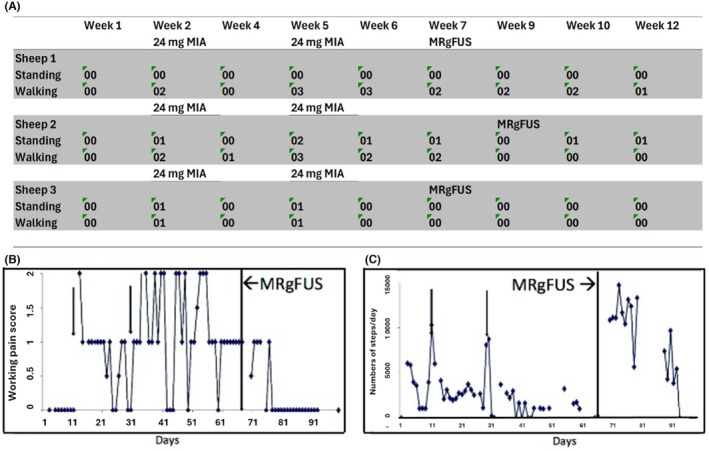
Pain scores and pedometer readings of sheep with monosodium iodoacetate (MIA)–induced knee osteoarthritis (KOA) treated with magnetic resonance–guided focused ultrasound (MRgFUS). (A) Table of pain scores for sheep when standing and when walking during the induction of MIA‐induced KOA and after treatment with MRgFUS. Standing scores: 0: continuous weight bearing, 1: intermittent weight bearing, 2: completely non‐weight bearing. Walking scores: 0: continuous weight bearing, 1: intermittent weight bearing, 2: toe‐touching/partial weight bearing, 3: non‐weight bearing. (B) Graph of the walking pain scores of sheep 2. (C) Pedometer data for sheep 2 capturing steps walked per day. (B, C) Arrows represent the timing of MIA‐intra‐articular injections inducing KOA, and vertical lines represent treatment using MRgFUS.

Despite consistent structural degeneration across animals, overt pain‐related behaviors were observed in only two of nine sheep. These animals developed a non‐weight‐bearing gait after MIA injection, reflected by elevated pain scores. One animal also exhibited a persistent non‐weight‐bearing posture of the affected limb. The remaining animals exhibited only transient changes in weight‐bearing and gait immediately after MIA injections, which were resolved within ~1 week (Figure 3A).

After MRgFUS treatment targeting the periarticular region of the affected knee, the two animals with persistent pain exhibited reduced pain scores that were maintained through the end of the study. In one animal, limping resolved completely within 10 days posttreatment (Figure 3B). In the subset of animals monitored using pedometers (*n* = 3), daily step counts increased after MRgFUS treatment. Particularly, step counts in one affected animal increased more than twofold after treatment, consistent with improved functional activity (Figure 3C).

### 3.4 MRgFUS treatment did not cause additional pain or detectable tissue damage

**FIGURE 4:**
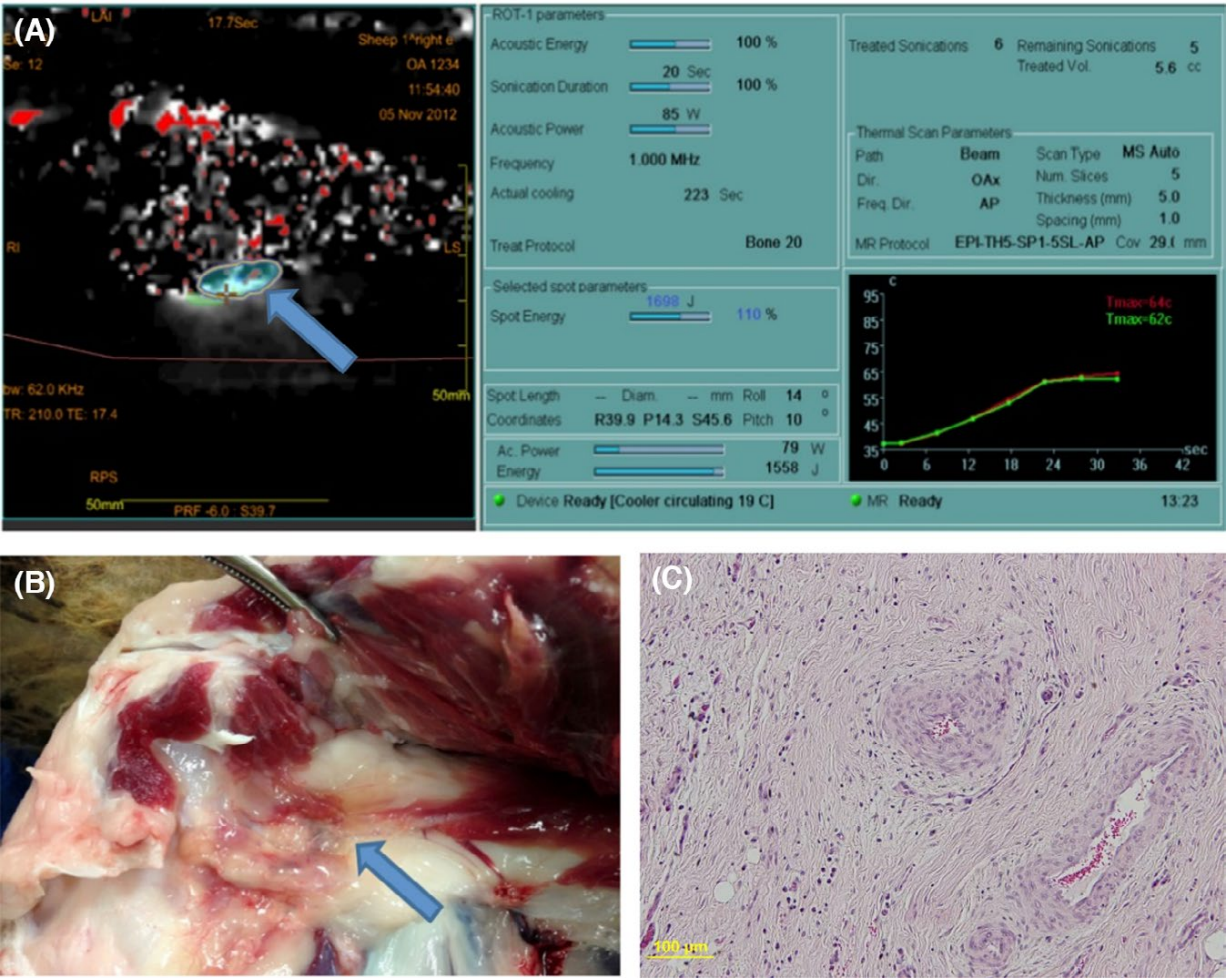
Magnetic resonance, fresh tissue, and histologic images after magnetic resonance–guided focused ultrasound (MRgFUS) therapy in sheep. (A) Thermal image and temperature graph demonstrating the location, temperature, and duration of ablation of the knee nerve supply. Ablation was focused on the region indicated by the arrow. (B) Gross pathologic examination and (C) H&E (hematoxylin and eosin) staining of the MRgFUS‐treated area demonstrating precise ablation of the target area and preservation of surrounding tissue.

MRgFUS treatment did not result in increased pain scores or new limping behavior in any animals, including those without preexisting pain. Animals without persistent pain prior to treatment maintained stable functional scores throughout the study period (Figure 3A). MR thermometry during treatment demonstrated localized thermal deposition at the targeted periarticular region (Figure 4A). Gross examination of treated joints and surrounding tissues showed no evidence of unintended tissue injury (Figure 4B). Histological evaluation using H&E staining further confirmed the absence of structural damage in tissues adjacent to the treatment site (Figure 4C).

## 4 DISCUSSION

In this study, we developed a large‐animal model of KOA in sheep using intra‐articular MIA injection. Sheep offer several advantages over commonly used small‐animal models. In particular, the anatomy of the sheep knee more closely resembles that of the human knee, and its larger size permits clinically relevant interventions such as arthroscopy and MRI, which are not feasible in rodent models.^10^, ^15^

A variety of methods exist to induce KOA across animal models; however, surgical approaches such as partial or total meniscectomy are most commonly used in sheep.^32^ Although meniscectomy reliably models post‐traumatic KOA and associated structural changes,^32^, ^33^ it is technically demanding and subject to intersurgeon variability. In contrast, chemically induced models such as intra‐articular MIA injection are simple to perform and may offer greater reproducibility.^11^, ^14^ MIA‐induced KOA is widely used in small‐animal models and is particularly valuable for studies of pain due to its relatively rapid and consistent induction of joint degeneration.^34^

The first objective of this study was to establish a sheep model of knee osteoarthritis using intra‐articular MIA injection. KOA was induced in the right hind knees of nine sheep using two intra‐articular injections of MIA administered 21 days apart. Radiographic analysis demonstrated joint space narrowing in the treated joints compared with contralateral controls. At the study endpoint, gross examination revealed cartilage degradation and exposure of subchondral bone, which were confirmed by histological analysis. Together, these findings indicate that intra‐articular MIA injection can reproducibly recapitulate the key structural features of KOA in a sheep model using a relatively simple and rapid approach.

KOA‐associated pain was assessed using a semiquantitative scoring system incorporating activity level, physical examination, and behavioral changes. Despite consistent structural degeneration, persistent pain‐related behaviors were observed in two of nine sheep. Prior studies in small‐animal models have demonstrated that the development of pain after MIA injection is dose dependent.^35^ The relatively low proportion of animals exhibiting sustained pain in this study suggests that the administered MIA dose may have been insufficient to consistently induce a robust pain phenotype in sheep. Future studies should optimize dosing and incorporate standardized radiographic and histological grading systems, such as the Kellgren–Lawrence scale and the Osteoarthritis Research Society International (OARSI) scoring, to enable quantitative comparisons across models.

The second aim of this study was to evaluate the feasibility, safety, and preliminary efficacy of MRgFUS for the treatment of KOA‐associated pain. Current nonsurgical management strategies for KOA include rehabilitation, NSAIDs, and intra‐articular corticosteroid injections.^6^ Although these approaches can provide symptomatic relief, many patients ultimately require joint replacement due to persistent pain and functional limitation.^5^, ^9^, ^36^ MRgFUS targeting periarticular nerve structures, including branches of the genicular nerves, represents a promising noninvasive alternative for pain management.

In this study, MRgFUS was applied to the periarticular region of the affected knee in all animals after MIA‐induced KOA. Among the two animals with persistent pain prior to treatment, improvements in pain scores and activity levels were observed after MRgFUS, including complete resolution of limping in one case. However, the small number of affected animals and the absence of a control group limit conclusions regarding treatment efficacy. These findings are preliminary and should be considered hypothesis generating due to the limited sample size and absence of controls.

Importantly, MRgFUS treatment showed no evidence of off‐target tissue injury on gross or histological evaluation. Furthermore, gross and histological evaluation of treated regions did not reveal evidence of damage to surrounding tissues. These findings suggest that MRgFUS is well tolerated in this model, although larger controlled studies are required to more definitively establish safety. This model may be particularly useful for evaluating device‐based and image‐guided interventions that require joint sizes comparable to those of humans.

This study has several limitations. First, the sample size was small, and no formal power calculation was performed, consistent with the exploratory nature of this pilot study. Second, no sham injection or sham MRgFUS control group was included, limiting causal interpretation of treatment effects. Third, although histological evaluation was performed, standardized scoring systems such as OARSI grading were not applied. Fourth, behavioral assessment relied on a semiquantitative scoring system and limited pedometer data, without quantitative gait or weight‐bearing analysis. Fifth, only two animals developed persistent pain, limiting conclusions regarding analgesic efficacy. Finally, the use of the contralateral limb as a control may introduce confounding due to compensatory biomechanical loading.

## 5 CONCLUSIONS

This study demonstrates that intra‐articular MIA injection can be used to establish a reproducible structural model of KOA in sheep that recapitulates the key structural features of the disease. In addition, MRgFUS treatment was well tolerated and exhibited preliminary potential for alleviating OA‐associated knee pain. These findings support further investigation of both this model and MRgFUS in larger, controlled studies.

## AUTHOR CONTRIBUTIONS

**Xuejun Du:** Data curation; writing – original draft; writing – review and editing. **Andrew Spiteri:** Writing – original draft; writing – review and editing. **Arik Hananel:** Data curation; writing – review and editing. **Abhijit Dighe:** Conceptualization; data curation; writing – review and editing. **Richard J. Price:** Conceptualization; writing – review and editing. **Xinlin Yang:** Supervision; validation; writing – original draft; writing – review and editing. **Quanjun Cui:** Funding acquisition; resources; writing – review and editing.

## FUNDING INFORMATION

This study was funded by the Focused Ultrasound Surgical Foundation and the Department of Orthopaedic Surgery at the University of Virginia.

## CONFLICT OF INTEREST STATEMENT

The authors declare that the study was conducted without any commercial or financial relationships that could be interpreted as a potential conflict of interest.

## ETHICS STATEMENT

This study was approved by the Institutional Animal Care and Use Committee at the University of Virginia (protocol no. 3933).

## Data Availability

The data that support the findings of this study are available from the corresponding author upon reasonable request.
